# The impact of fine particulate matter (PM) on various beneficial functions of human endometrial stem cells through its key regulator SERPINB2

**DOI:** 10.1038/s12276-021-00713-9

**Published:** 2021-12-02

**Authors:** Se-Ra Park, Joong Won Lee, Seong-Kwan Kim, Wook-Joon Yu, Seung-Jin Lee, Doojin Kim, Kun-Woo Kim, Ji-Won Jung, In-Sun Hong

**Affiliations:** 1 Department of Health Sciences and Technology, GAIHST, Gachon University, Incheon, 21999 Republic of Korea; 2 Department of Molecular Medicine, School of Medicine, Gachon University, Incheon, 406-840 Republic of Korea; 3 Division of Allergy and Chronic Respiratory Diseases, Center for Biomedical Sciences, Korea National Institute of Health, Korea Centers for Disease Control and Prevention, Cheongwon-gun, Republic of Korea; 4 Developmental and Reproductive Toxicology Research Group, Korea Institute of Toxicology, Deajeon, 34114 Republic of Korea; 5 Department of Surgery, Gachon University Gil Medical Center, Gachon University School of Medicine, Incheon, Republic of Korea; 6 Department of Thoracic and Cardiovascular Surgery, Gachon University Gil Medical Center, Incheon, Republic of Korea

**Keywords:** Stem-cell research, Adult stem cells

## Abstract

Fine particulate matter (PM) has a small diameter but a large surface area; thus, it may have broad toxic effects that subsequently damage many tissues of the human body. Interestingly, many studies have suggested that the recent decline in female fertility could be associated with increased PM exposure. However, the precise mechanisms underlying the negative effects of PM exposure on female fertility are still a matter of debate. A previous study demonstrated that resident stem cell deficiency limits the cyclic regenerative capacity of the endometrium and subsequently increases the pregnancy failure rate. Therefore, we hypothesized that PM exposure induces endometrial tissue damage and subsequently reduces the pregnancy rate by inhibiting various beneficial functions of local endometrial stem cells. Consistent with our hypothesis, we showed for the first time that PM exposure significantly inhibits various beneficial functions of endometrial stem cells, such as their self-renewal, transdifferentiation, and migratory capacities, in vitro and in vivo through the PM target gene SERPINB2, which has recently been shown to be involved in multiple stem cell functions. In addition, the PM-induced inhibitory effects on the beneficial functions of endometrial stem cells were significantly diminished by SERPINB2 depletion. Our findings may facilitate the development of promising therapeutic strategies for improving reproductive outcomes in infertile women.

## Reproductive health: Pollution is problematic for female fertility

Airborne pollutants may reduce female fertility through their debilitating effects on the stem cells that maintain the endometrium, the interior lining of the uterus. Recent evidence suggests that toxic byproducts from fossil fuels known as ‘particulate matter’ represent a danger to women’s reproductive health. South Korean researchers led by Ji-Won Jung, Korea Centers for Disease Control and Prevention, and In-Sun Hong, Gachon University, Incheon, have investigated this risk by exposing cultured human endometrial stem cells to diesel-derived particulate matter. These stem cells normally maintain the endometrium, allowing embryonic implantation to take place, but exposure to particulate matter greatly impaired the cells’ regenerative function. Mice exposed to particulate matter exhibited similar impairments of endometrial maintenance. The researchers identified a molecular pathway associated with this response that could guide development of fertility-restoring treatments.

## Introduction

Recently, accelerated industrialization and urban development have worsened air pollution, and its harmful effects have become major health concerns in many developed countries^1,2^. Among the various air pollutants, fine particulate matter (PM), which is a mixture of multiple particles, including organic compounds, toxic metals, and crustal elements from different sources^3,4^, is a serious international health concern due to its close link to various deleterious effects of air pollution on human health^5,6^. Fine PM (with a maximum diameter of 2.5 μm) has a small diameter but a large surface area; thus, it might have widespread toxic effects that subsequently damage many tissues of the human body^5^. Although there is currently no evidence that PM is directly detected in human reproductive organs such as the uterus and ovaries, various studies have suggested that PM can negatively affect reproductive organs and the subsequent pregnancy rate. Indeed, Perin et al. revealed that early pregnancy loss can be associated with acute maternal exposure to high levels of PM^7^. Similarly, women exposed to high concentrations of PM during the follicular phase of the menstrual cycle may have an increased risk of miscarriage compared to that of women exposed to low concentrations^8^. In this context, few studies have attempted to explain how PM exposure can trigger female infertility and increase the rate of pregnancy loss^9,10^. Furthermore, the precise mechanisms underlying PM exposure-related female infertility are still poorly understood, probably due to the multiplicity of factors affecting female reproductive function and fertility^11–13^.

The uterine endometrium is one of the most dynamically remodeled human tissues, as it undergoes rapid growth of up to 7 mm per week during the regular menstrual cycle before embryo implantation^14^. Successful embryo implantation in a receptive uterine endometrium requires constant activation and recruitment of local stem cells that can properly differentiate into various endometrial cell types^15^. Interestingly, a recent study found that accelerated cellular aging of local endometrial stem cells reduces the regenerative capacity of the uterine endometrium and subsequently decreases the rate of successful pregnancy outcomes^15^. In this context, it is reasonable to hypothesize that PM exposure may induce endometrial tissue damage and subsequently reduce the rate of favorable pregnancy outcomes by inhibiting various functions of local endometrial stem cells. Unfortunately, to the best of our knowledge, there are few data available on the inhibitory effects of PM exposure on local stem cells, with results reported for only a few tissues. For example, the number of bone marrow-derived endothelial progenitor cells (EPCs) is significantly reduced and the function of these cells is significantly suppressed in animals exposed to PM^16,17^. Based on these limited results, it is currently difficult to conclude whether these reductions in stem cell populations or functions within a tissue are due to the direct inhibitory effects of PM exposure or to indirect PM-induced effects, such as systemic formation of reactive oxygen species (ROS)^18^ and inflammatory responses^19^.

Consistent with our hypothesis, we show for the first time that PM exposure significantly inhibits various beneficial functions of endometrial stem cells, such as their aging, self-renewal, transdifferentiation, and migratory capacities, in vitro and in vivo. We subsequently explored the molecular mechanism underlying these PM-induced inhibitory effects on endometrial stem cells. Strikingly, PM exposure markedly increased the expression of SERPINB2, which was recently found to be involved in multiple stem cell functions^20,21^. Importantly, the PM-induced inhibitory effects on various stem cell functions were markedly attenuated by SERPINB2 depletion, suggesting that SERPINB2 may act as a key mediator of PM-induced inhibitory effects in endometrial stem cells. Taken together, the results of the present study provide valuable information and reveal novel underlying mechanisms activated in response to exposure to PM or other air pollutants, and the data will help to identify strategies for preventing or treating infertility related to PM exposure.

## Materials and methods

### Isolation and culture of human endometrial stem cells from uterine tissues

Human endometrial stem cells were obtained from endometrial tissues of uterine fibroid patients with written informed consent from the patients and approval of the Gachon University Institutional Review Board (IRB No: GAIRB2018-134). To isolate human endometrial stem cells, endometrial tissue from women undergoing hysterectomy for treatment of uterine fibroids was minced into small pieces, and the small pieces were then digested in DMEM containing 10% FBS and 250 U/ml type I collagenase for 5 h at 37 °C in a rotating shaker. The digestion mixture was then filtered through a 40 µm cell strainer to separate spindle-shaped endometrial stem cells from epithelial gland fragments and undigested tissue. Endometrial stromal cells were removed from the endometrial cells that were passed through the cell strainer by quick attachment (within 30 min) to a cell culture dish. Unattached endometrial stem cells were harvested and were then cultured in EBM-2 medium (Lonza) supplemented with EGM-2 at 37 °C in 5% CO_2_^5^.

### Cell proliferation assay

An MTT assay was used to determine the antiproliferative capacity of PM (diesel particulate matter; SRM® 1650b; NIST, Sigma–Aldrich, USA) exposure. Briefly, cells (1 × 10^4^ cells/well) were seeded in 48-well plates in EBM-2 medium (Lonza) supplemented with EGM-2. After 24 h of incubation, the plates were washed with PBS, and the cells were then treated with PM or vehicle for 72 h in serum and supplement-free EBM-2 medium. Thereafter, 50 µl of MTT solution (Sigma–Aldrich, M5655, 5 mg/ml in PBS) was added to each well, and the cells were incubated at 37 °C for another 3 h. Cell viability was assessed by measuring the absorbance at 570 nm using a VersaMax microplate reader.

### Senescence-associated beta-galactosidase (SA β-gal) staining

SA β-gal staining was performed as previously described^22^. Endometrial stem cells were seeded in 6-well plates at a density of 1 × 10^5^ cells/well. The cells were incubated for 3 days to the appropriate confluence. The cells were then washed twice with PBS and fixed with 0.5% glutaraldehyde in PBS for 5 min. The cells were then washed with PBS containing 1 mM MgCl_2_ and stained with X-gal solution [1 mg/ml X-gal, 0.12 mM K_3_Fe(CN)_6_, 1 mM MgCl_2_ in PBS (pH 6.0)] overnight at 37 °C.

### In vitro cell migration assay

Cells were plated at 1 × 10^5^ cells/well in 200 μL of culture medium in the upper chambers of Transwell permeable supports (Corning Inc., Corning, NY, USA) to track the migration of cells. The Transwell chambers had 6.5-mm-diameter polycarbonate membranes with an 8.0 μm pore size in a 24-well plate format. Noninvaded cells on the upper surface of each membrane were removed by scrubbing with a Kimwipe. Migrated cells on the lower surface of each membrane were fixed with 4% paraformaldehyde for 5 min and stained with hematoxylin for 15 min. Later, the number of migrated cells was counted in three randomly selected fields of view for each well under a light microscope at 50X magnification. To calculate the chemotactic index, the number of cells that migrated in response to PM treatment was divided by the number of spontaneously migrating cells.

### Real-time PCR

Total RNA was extracted using TRIzol reagent (Invitrogen) according to the manufacturer’s protocol. RNA purity was verified by measuring the 260/280 absorbance ratio. First-strand cDNA was synthesized from 1 μg of total RNA using SuperScript II (Invitrogen), and one-tenth of the cDNA was added to each PCR mixture containing Express SYBR-Green qPCR Supermix (BioPrince, Seoul, South Korea). Real-time PCR was performed using a Rotor-Gene Q thermocycler (Qiagen). PCR was performed with 40 cycles of amplification with denaturation at 95 °C for 20 s, annealing at 60 °C for 20 s and extension at 72 °C for 25 s. The relative mRNA expression of the selected genes was normalized to that of PPIA and quantified using the ΔΔCT method. The sequences of the PCR primers are listed in Table 1.

**Table 1:** Quantitative RT–PCR primer sequences.

Gene	RefSeq accession no.	Direction	Primer sequence
Human PPIA	NM_021130	F	TGCCATCGCCAAGGAGTAG
		R	TGCACAGACGGTCACTCAAA
Human IL6	NM_000600	F	GGTACATCCTCGACGGCATCT
		R	GTGCCTCTTTGCTGCTTTCAC
Human P16	NM_000077	F	CTACTGAGGAGCCAGCGTCT
		R	CTGCCCATCATCATGACCT
Human P18	NM_001262	F	TGGGTCTTCCGCAAGAACTC
		R	TGGCAGCCAAGTGCAAGGGC
Human P21	NM_000389	F	ACAGCAGAGGAAGACCATGTGGACC
		R	CGTTTTCGACCCTGAGAGTCTCCAG
Human C-MYC	NM_002467	F	AAAGGCCCCCAAGGTAGTTA
		R	GCACAAGAGTTCCGTAGCTG
Human KLF4	NM_001314052	F	GAACTGACCAGGCACTACCG
		R	TTCTGGCAGTGTGGGTCATA
Human NANOG	NM_024865	F	TGGGATTTACAGGCGTGAGC
		R	AAGCAAAGCCTCCCAATCCC
Human OCT4	NM_002701	F	AGCCCTCATTTCACCAGGCC
		R	TGGGACTCCTCCGGGTTTTG
Human SOX2	NM_003106	F	AAATGGGAGGGGTGCAAAAGAGGAG
		R	CAGCTGTCATTTGCTGTGGGTGATG
Human SERPINB2	NM_001143818	F	ACCCCCATGACTCCAGAGAACT
		R	GAGAGCGGAAGGATGAATGGAT
Mouse HPRT	NM_013556	F	GCCTAAGATGAGCGCAAGTTG
		R	TACTAGGCAGATGGCCACAGG
Mouse C-MYC	NM_010849	F	CGCACACACAACGTCTTGGA
		R	AGGATGTAGGCGGTGGCTTT
Mouse KLF4	NM_010637	F	GGTGCAGCTTGCAGCAGTAA
		R	AAAGTCTAGGTCCAGGAGGT
Mouse NANOG	NM_028016	F	GCCTTACGTACAGTTGCAGC
		R	TCACCTGGTGGAGTCACAGA
Mouse OCT4	NM_013633	F	GCATTCAAACTGAGGCACCA
		R	AGCTTCTTTCCCCATCCCA
Mouse SOX2	NM_011443	F	GAAGCGTGTACTTATCCTTCTTCAT
		R	GAGTGGAAACTTTTGTCCGAGA

### Protein isolation and western blot analysis

Protein expression levels were determined by western blot analysis as previously described^23^. Cells were lysed in a buffer containing 50 mM Tris, 5 mM EDTA, 150 mM NaCl, 1 mM DTT, 0.01% NP 40, and 0.2 mM PMSF. The protein concentrations in the total cell lysates were measured by using bovine serum albumin as the standard. Samples containing equal amounts of protein were separated via sodium dodecyl sulfate-polyacrylamide gel electrophoresis (SDS–PAGE), and proteins were then transferred onto nitrocellulose membranes (Bio–Rad Laboratories). Membranes were blocked with 5% skim milk in Tris-buffered saline containing Tween 20 at RT. Then, the membranes were incubated first with primary antibodies against β-actin (Abcam, MA, USA, ab189073), MMP-2 (Cell Signaling #4022), MMP-9 (Cell Signaling #13667), SERPINB2 (Abcam, MA, USA, ab47742), and caspase-3 (Cell Signaling, MA, USA, #9662) overnight at 4 °C and then with HRP-conjugated goat anti-rabbit IgG (BD Pharmingen, San Diego, CA, USA, 554021) and goat anti-mouse IgG (BD Pharmingen, 554002) secondary antibodies for 60 min at RT. Antibody-bound proteins were detected using ECL reagents.

### Immunofluorescence staining

Samples were fixed with 4% paraformaldehyde for fluorescence staining. Samples were permeabilized with 0.4 M glycine and 0.3% Triton X-100, and nonspecific binding was blocked with 2% normal swine serum (DAKO, Glostrup, Denmark). Staining was performed as described previously^24^ using an anti-phalloidin (Cytoskeleton Inc.) primary antibody. Samples were examined by fluorescence microscopy (Zeiss LSM 510 Meta). Expression was calculated based on the area and density of red fluorescence divided by the number of cells, as determined from the number of DAPI-stained nuclei, in three randomly selected fields of view for each sample from a total of three independent experiments.

### Osteogenic differentiation

Endometrial stem cells were incubated in high-glucose DMEM supplemented with 0.1 µM dexamethasone, 10 mM β-glycerophosphate, 50 µM ascorbate and 10% FBS and were treated with or without PM exposure. Endometrial stem cells were grown for 3 weeks, with medium replacement twice a week. Differentiated cells were stained with Alizarin Red S to detect de novo formation of bone matrix. Alizarin red S staining in the samples was quantified by measuring the optical density (OD) of the solution at 570 nm.

### Adipogenic differentiation

Endometrial stem cells were incubated in low-glucose DMEM supplemented with 500 µM methylxanthine, 5 µg/mL insulin, and 10% FBS and were treated with or without PM exposure. Endometrial stem cells were grown for 3 weeks, with medium replacement twice a week. Lipid droplet formation was confirmed by Oil Red O staining. Relative quantification of lipid droplet formation was determined by measuring the absorbance at 500 nm.

### Analysis of mitochondrial respiration and glycolytic capacity

With a Seahorse XF analyzer (Seahorse Bioscience, North Billerica, MA), mitochondrial oxidative phosphorylation and glycolytic flux can be analyzed in real time by measuring the OCR and ECAR of cells as they respond to substrates and metabolism-inhibiting agents according to the manufacturer’s instructions^25^. The ATP synthase inhibitor oligomycin (a complex V blocker) is added to inhibit ATP-coupled respiration. FCCP (a mitochondrial uncoupler) is added to collapse the mitochondrial membrane potential (Δψm). Rotenone (an inhibitor of complex I in the electron transport chain) and antimycin A (an inhibitor of complex III in the electron transport chain) are added to block mitochondrial respiration completely. To measure real-time glycolytic rates, the Seahorse XF glycolytic rate assay utilizes both extracellular acidification rate (ECAR) and OCR measurements to evaluate the glycolytic proton efflux rate (glycoPER) of the cells; in this assay, cells are incubated in glucose-free medium to which rotenone, antimycin A, and finally 2-deoxyglucose (2-DG, a glycolysis inhibitor) are sequentially added. The OCR and ECAR were described as absolute rates (pmoles/min for OCR and mpH/min for ECAR) and normalized to the cell number as a percentage of the baseline oxygen consumption.

### SERPINB2 knockdown

Small hairpin RNA (shRNA; accession no. NM_002575) targeting SERPINB2 and scrambled shRNA (shCon) were purchased from Bioneer (Daejeon, South Korea). For efficient SERPINB2 transfection, reverse transfection was performed using Lipofectamine 2000 (Invitrogen) according to the manufacturer’s protocol. Briefly, shRNA targeting SERPINB2 (3 μg/ml) was mixed with 3 μl of the transfection reagent Lipofectamine 2000 in Gibco Opti-MEM medium without FBS and antibiotics. Five hours before transfection, the Opti-MEM was replaced with fresh EGM-2 supplemented with 10% FBS. We chose the SERPINB2 shRNA that was most effective at the mRNA level from three shRNAs designed from the target sequence and analyzed by qRT–PCR.

### Flow cytometry

FACS analysis and cell sorting were performed using FACS Calibur and FACS Aria instruments (Becton Dickinson, Palo Alto, CA), respectively. FACS data were analyzed using FlowJo software (Tree Star, Ashland, OR). Antibodies against the following proteins were used: PE-conjugated CD34 (MACS; Miltenyi Biotech, 30-081-002), CD44 (MACS; Miltenyi Biotech, 130-095-180), CD45 (MACS; Miltenyi Biotech, 130-080-201), CD73 (MACS; Miltenyi Biotech, 130-095-182), CD105 (MACS; Miltenyi Biotech, 130-094-941), CD140b (MACS; Miltenyi Biotech, 130-105-279), and W5C5 (MACS; Miltenyi Biotech, 130-111-641). The FACS gates were established by staining with an isotype antibody or secondary antibody.

### Growth factor antibody array assay

The assay was performed following the manufacturer’s protocol (Abnova AA0089). Briefly, were incubated with antibody-spotted membranes were incubated with PM- or vehicle-treated protein samples overnight at 4 °C. After washing 3 times with wash buffer, the membranes were incubated with biotin-conjugated anti-cytokine antibodies overnight at 4 °C. The membranes were then washed 3 times and incubated with HRP-conjugated streptavidin. Chemiluminescence was used to detect signals from the growth factor antibodies spotted on the nitrocellulose membrane.

### Gene Expression Omnibus (GEO) database analysis

Gene Expression Omnibus (GEO) (https://www.ncbi.nlm.nih.gov/geo/) is a freely distributed database of high-throughput gene expression data generated by genome hybridization array, chip sequencing and DNA microarray analyses^26,27^. Researchers provide their experimental results in four categories: experimental designs, samples, platforms, and raw data. Clinical or experimental samples within each dataset are further organized based on various experimental subgroups, such as treatment, physiologic condition, and disease state. These categorized biological data are presented as a “GEO profile”, which includes the dataset title, gene annotation, a chart depicting the expression levels, and the rank for that gene across each sample^28^. The expression profiles of AR, EGF, FGF4/7, HGF, IGFBP4/6, NT-4, PDGFR β, TGF-β3, and VEGFR3 in response to various toxic exposures were analyzed according to previously established procedures^28^.

### Ingenuity pathway analysis

Analysis of SERPINB2-related genes was performed with Ingenuity Pathway Analysis (IPA) version 2.0 software (Ingenuity Systems, Redwood City, CA). Differentially expressed genes (*t*-test, *P* < 0.005) between toxicant-treated cells and untreated cells were subjected to analysis of SERPINB2-related genes (GSE69851). The significance of each molecule was evaluated by Fisher’s exact test (*P* value), which was used to identify the differentially expressed genes identified by microarray analysis that overlapped with genes known to be regulated by a molecule. The activation score (Z-score) was used to show the status of predicted molecules by comparing the observed differential regulation of the gene (“up” or “down”) in the microarray data relative to the literature-derived regulation direction, which could be either activating or inhibiting.

### GeneMANIA algorithm-based bioinformatics analysis

To further analyze eleven genes that were involved in or directly regulated self-renewal, pluripotency, and migratory capacity, we imported all identified genes and their corresponding accession numbers into GeneMANIA (http://www.genemania.org). To identify gene interactions, we considered several factors, including coexpression, colocalization, and genetic interactions. From this list, we selected the AR, EGF, FGF4/7, HGF, IGFBP4/6, NT-4, PDGFR β, TGF-β3, and VEGFR3 genes to test their involvement in regulating various cellular functions, such as self-renewal, pluripotency, and migratory capacity.

### Evaluation of the effects of PM exposure in an animal model

All of the animal experiments were approved by and conducted in accordance with the guidelines of the Institutional Animal Care and Use Committee (KCDC-029-20-2A) of the Korea Centers for Disease Control and Prevention. Mice were randomly divided into the control (vehicle), PM low (20 mg/kg) and PM high (40 mg/kg) treatment groups. ICR mice were exposed to PM or vehicle (PBS) two times via intraperitoneal injection. Mice were anesthetized and exsanguinated by cardiac puncture, and endometrial stem cells were then isolated from the uterine endometrium.

### Statistical analysis

All statistical data were analyzed in GraphPad Prism 5.0 (GraphPad Software, San Diego, CA) and evaluated using two-tailed Student’s *t* tests. Values of *P* < 0.05 were considered to indicate statistical significance.

## Results

### PM exposure markedly inhibits various beneficial functions of human endometrial stem cells

**Fig. 1:**
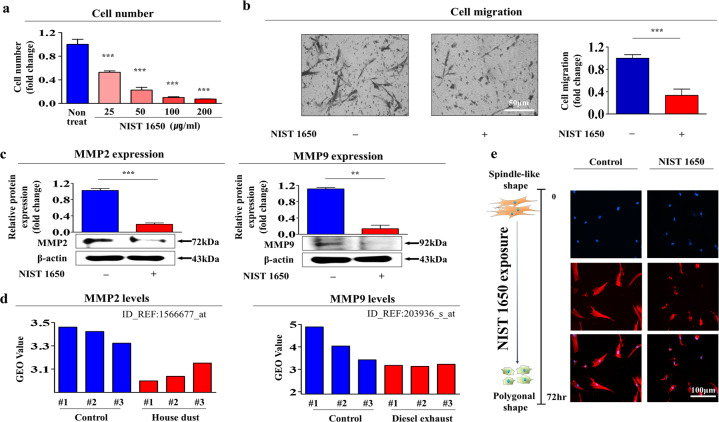
PM exposure significantly inhibits the self-renewal and reduces the migratory ability of endometrial stem cells in vitro. We hypothesized that PM exposure inhibited various beneficial functions of endometrial stem cells, such as self-renewal, transdifferentiation, and migration. The inhibition of endometrial stem cell self-renewal by treatment with various concentrations of PM (diesel particulate matter, NIST 1650; 25, 50, 100, and 200 μg/ml) was evaluated at 72 h by an MTT assay. Stem cell viability (%) was calculated as a percent of the viability of cells treated with vehicle control (**a**). Endometrial stem cells were treated with PM (25 μg/ml) for 72 h, and the inhibitory effect of PM exposure on the stem cell migratory capacity was then analyzed using a Transwell migration assay. PM exposure markedly reduced the ability of cells to migrate across the membrane compared with that of control cells (**b**). The relative expression of important regulators of cell migration (MMP-2/9) with and without PM exposure was evaluated by western blot analysis (**c**). The GEO database was further analyzed to verify the correlations between increased expression levels of MMP-2/9 and exposure to various air pollutants (**d**). PM-induced morphological changes and dynamic actin filament disorganization in endometrial stem cells were analyzed by staining actin filaments with phalloidin (**e**). DAPI was used to label nuclei. β-Actin was used as the internal control. The bar graphs show the average of three independent experiments. Significant differences are presented. *p < 0.05, **p < 0.005, and ***p < 0.001 (two-sample *t*-test).

To evaluate the effects of PM exposure, we isolated human endometrial stem cells from uterine tissue (Supplementary Fig. 1a) and then assessed their biological characteristics by analyzing several positive or negative surface markers of stem cells. Four positive surface markers (CD44, CD73, CD105, and CD140b) were expressed on most (more than 99%) cells, while only a certain percentage (65–69%) of cells expressing two positive surface markers (CD146 and W5C5) were detected in the total cell population (Supplementary Fig. 1b). Therefore, it can be postulated that endometrial stem cells isolated from endometrial tissues are a heterogeneous population of cells containing at least two types of cells. In addition, the differentiation potential of endometrial stem cells into other cell types was assessed by inducing adipogenesis and osteogenesis in vitro (Supplementary Fig. 1c). The results suggest that endometrial stem cells isolated from endometrial tissues may be a heterogeneous cell population but possess a clear transdifferentiation capacity as stem cells. Next, we evaluated whether PM (diesel particulate matter, NIST 1650) exposure inhibited the multiple beneficial functions of endometrial stem cells in vitro. Importantly, we found a steady decline in the self-renewal capacity of endometrial stem cells in response to PM exposure (Fig. 1a). PM treatment also significantly decreased the self-renewal capacity of endometrial stem cells in a time-dependent manner (Supplementary Fig. 2). Interestingly, Transwell assays showed the inhibitory effect of PM exposure on the migration of endometrial stem cells (Fig. 1b). To further evaluate the suppressive effect of PM on the migration of stem cells, western blotting was used to measure the expression levels of matrix metalloproteinase-2/9 (MMP-2/9), which play regulatory roles in cell migration (Fig. 1c). Similarly, GEO dataset analysis revealed that MMP-2/9 expression was also clearly increased in response to exposure to air pollutants such as home dust and fine particulate matter (Fig. 1d). Our previous results revealed that actin filament rearrangement (polymerization/depolymerization) can lead to the “pushing” and “pulling” of stem cells during their migration^29^. Consistent with this observation, phalloidin staining of the actin cytoskeleton revealed a strong correlation between PM exposure and increased filament depolymerization (Fig. 1e), indicating that the migration inhibition of PM-exposed stem cells may be associated with actin filament disorganization.

**Fig. 2:**
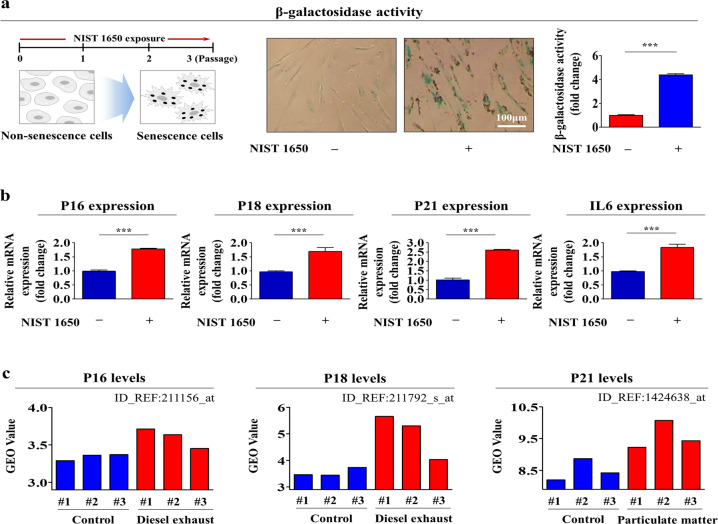
PM exposure markedly induces senescence in endometrial stem cells in vitro. The effects of PM exposure on endometrial stem cell aging in vitro were analyzed by evaluating senescence-associated β-galactosidase (SA-β-Gal) activity. Endometrial stem cells were pretreated with PM (25 μg/ml) for 72 h, and changes in stem cell aging were determined by evaluating SA-β-Gal activity (**a**). The effects of PM exposure on the expression of multiple cellular senescence markers (p16, p18, p21, and IL-6) were evaluated by real-time PCR (**b**). The Gene Expression Omnibus (GEO) database (https://www.ncbi. nlm.nih.gov/geo/) was used to further analyze the correlations between increased expression levels of these senescence markers and exposure to various air pollutants (**c**). The bar graphs show the average of three independent experiments. Significant differences are presented. *p < 0.05, **p < 0.005, and ***p < 0.001 (two-sample *t*-test).

The most commonly exploited characteristic of cellular aging is the enhanced enzymatic activity of senescence-associated β-galactosidase (SA-β-Gal)^30^. Therefore, to evaluate whether PM exposure accelerates cellular aging, endometrial stem cells were continuously subcultured with or without exposure to PM (Fig. 2a). In addition to increased SA-β-Gal activity, enhanced levels of cytoplasmic proteins, such as p16, p18, p21, and IL-6, have been widely used as surrogate markers of cellular aging^31^. The levels of these cellular aging-associated markers were markedly enhanced by PM exposure (Fig. 2b). In addition, we further analyzed the Gene Expression Omnibus (GEO) database to determine the correlations between the levels of these senescence markers and exposure to various types of air pollution. Indeed, the levels of these senescence markers were markedly increased in response to exposure to air pollutants such as diesel exhaust and fine particulate matter (Fig. 2c).

**Fig. 3:**
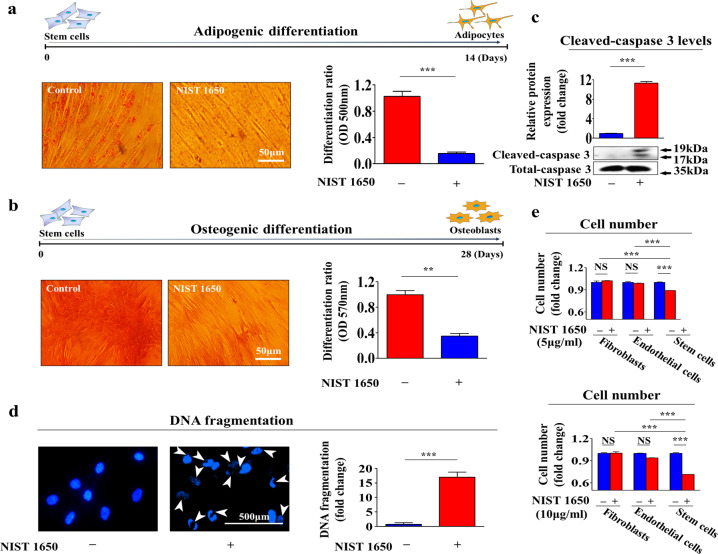
PM exposure markedly suppresses the multilineage differentiation potential and induces the apoptosis of endometrial stem cells in vitro. Endometrial stem cells were cultured in adipocyte or osteoblast differentiation medium with or without PM (25 μg/ml). The inhibitory effects of PM exposure on adipocyte (**a**) and osteoblast (**b**) differentiation were evaluated by oil red O and alizarin red staining, respectively. The relative quantification of calcium mineral content and lipid droplet formation in differentiated cells was performed by measuring the absorbance at 500 nm and 570 nm, respectively. Endometrial stem cells were cultured with or without PM (25 μg/ml) for 72 h, and the increase in the level of cleaved caspase-3 following PM exposure was then analyzed by western blotting (**c**). PM-induced apoptotic DNA condensation and fragmentation after incubation in the absence of PM (25 μg/ml) for 72 h were analyzed using DAPI staining (**d**). The decrease in cell viability following PM exposure (5 and 10 μg/ml) for 72 h was detected via an MTT assay in both endometrial stem cells and terminally differentiated cells (fibroblasts and vascular endothelial cells). Cell viability (%) was calculated as the percent of the viability of cells treated with the vehicle control (**e**). DAPI was used to label nuclei. β-Actin was used as the internal control. The bar graphs show the average of three independent experiments. Significant differences are presented. *p < 0.05, **p < 0.005, and ***p < 0.001 (two-sample *t*-test).

**Fig. 4:**
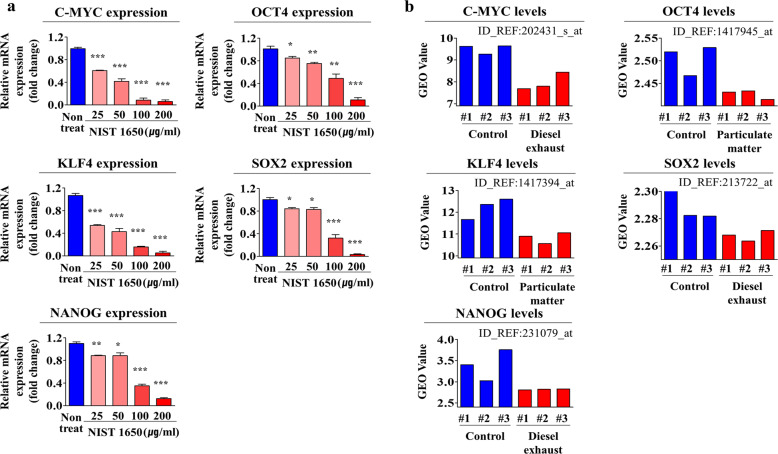
PM exposure reduces the expression of various stem cell markers in endometrial stem cells in vitro. The inhibitory effects of PM exposure on the expression of the stem cell markers C-MYC, KLF4, NANOG, OCT4, and SOX2 were evaluated by real-time PCR (**a**). The GEO database was also analyzed to further verify the correlations between increased expression levels of these pluripotency-associated factors and exposure to air pollutants (**b**). The bar graphs show the average of three independent experiments. Significant differences are presented. *p < 0.05, **p < 0.005, and ***p < 0.001 (two-sample *t*-test).

More strikingly, PM exposure markedly reduced the capacity of the cells to differentiate into adipocytes (Fig. 3a) and osteoblasts (Fig. 3b) in vitro. In addition, PM exposure increased the activity of the proapoptotic protein caspase-3 (Fig. 3c) and resulted in subsequent apoptosis, as indicated by DNA fragmentation (Fig. 3d). Importantly, we found that PM treatment more profoundly affected the self-renewal capacity of stem cells than of terminally differentiated somatic cells, such as dermal fibroblasts and vascular endothelial cells (Fig. 3e). The expression levels of the pluripotency-associated factors C-MYC, KLF4, NANOG, OCT4, and SOX2 were also significantly decreased by PM exposure (Fig. 4a). Consistent with these findings, GEO dataset analysis revealed that the expression levels of these pluripotency-associated factors were decreased in response to exposure to air pollutants such as diesel exhaust and fine particulate matter (Fig. 4b). These results suggest that PM exposure inhibits various beneficial functions, such as self-renewal, transdifferentiation, and migration, of endometrial stem cells.

### Metabolic activities (mitochondrial oxidative phosphorylation and glycolysis) are significantly enhanced by PM exposure

**Fig. 5:**
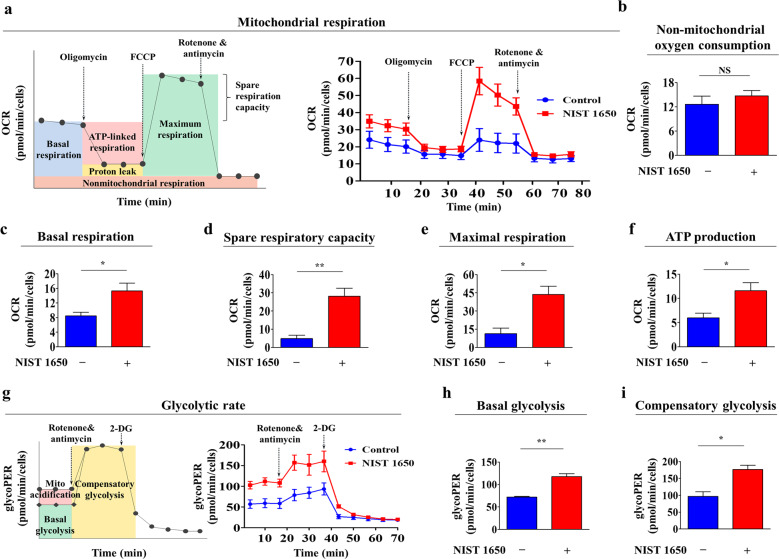
PM exposure significantly elevates the mitochondrial respiratory and glycolytic rates in endometrial stem cells. To evaluate the effect of PM exposure on the bioenergetic status of endometrial stem cells, the mitochondrial respiration and glycolytic rates were analyzed with or without PM exposure (**a**). Respiratory flux profiles of endometrial stem cells with or without exposure to PM (25 μg/ml) for 72 h were determined by collecting twelve consecutive oxygen consumption rate (OCR) measurements using a Seahorse XF analyzer. Cells were detached using trypsin-EDTA and reseeded in licensed cell culture miniplates at a density of 2 × 10^4^ cells per well in complete growth medium supplemented with 10% FBS. Cells were incubated overnight and washed 3 times with Seahorse XF medium. The ATP synthase inhibitor oligomycin (a complex V blocker, 1.5 μM) was added to inhibit ATP-coupled respiration. FCCP (a mitochondrial uncoupler, 2 μM) was added to decrease the mitochondrial membrane potential (Δψm). Rotenone (an inhibitor of complex I in the electron transport chain, 0.5 µM) and antimycin A (an inhibitor of complex III in the electron transport chain, 0.5 µM) were added to block mitochondrial respiration completely. The inhibitors were applied automatically in the analyzer, and the OCR was measured at 15-min intervals (**b**). PM exposure significantly increased the basal respiration rate (**c**), spare respiratory capacity (**d**), and maximal respiration rate (**e**) in endometrial stem cells. Overall ATP production in endometrial stem cells was significantly enhanced by PM exposure (**f**). The schematic of the procedure for real-time analysis of glycolysis in endometrial stem cells using the Seahorse XF analyzer is shown (**g**). For real-time analysis of glycolytic rates, the Seahorse XF glycolytic rate assay utilizes both ECAR (extracellular acidification rate) and OCR measurements to evaluate the glycolytic proton efflux rate (glycoPER) of the cells; in this assay, cells are incubated in glucose-free medium to which rotenone, antimycin A (1.67 μM), and finally 2-deoxyglucose (2-DG, glycolysis inhibitor, 50 mM) are sequentially added. The inhibitors were applied automatically in the analyzer, and the ECAR was measured at 15-minute intervals. The percentage of PER from glycolysis represents the contribution of the glycolytic pathway to the total ECAR (**h**). Compensatory glycolysis is the rate of glycolysis in cells following the inhibition of oxidative metabolism and refers to the ability of cells to drive compensatory energy production by using glycolysis to meet their energy demands (**i**). The ECAR values were normalized to the number of cells in each well. The bar graphs show the average of three independent experiments. Significant differences are presented. *p < 0.05, **p < 0.005, and ***p < 0.001 (two-sample *t*-test).

Metabolic changes regulate various stem cell behaviors (e.g., self-renewal, migration, and differentiation) by modulating energy (ATP) production through oxidative phosphorylation and/or glycolysis^32–34^. In addition, toxic exposure also induces alterations in various metabolic phenotypes in multiple cell types^35,36^. The rate of mitochondrial oxidative phosphorylation is one of the reliable indicators of cellular ATP production capacity and overall cellular health^37^. In this context, to evaluate the effect of PM exposure on the bioenergetic status of endometrial stem cells (Fig. 5a), mitochondrial respiration assays were performed using an Agilent Seahorse XF analyzer, which provides accurate real-time measurement of the oxygen consumption rate (OCR) in living cells^38^. To inhibit ATP-coupled respiration in mitochondria, the ATP synthase inhibitor oligomycin (a complex V blocker) was used to treat cells. Treatment with FCCP (a mitochondrial uncoupler) decreases the mitochondrial membrane potential (Δψm), leading to rapid consumption of oxygen without ATP production by inducing direct transport of protons across the inner mitochondrial membrane^37^. Therefore, the addition of FCCP allows the estimation of the maximum rate of respiration that the cell can achieve. The overall mitochondrial respiratory response of endometrial stem cells was significantly elevated by PM exposure, while the level of nonmitochondrial oxygen consumption was not changed (Fig. 5b). Consistent with these results, PM exposure also markedly increased the basal respiration rate (Fig. 5c), spare respiratory capacity (Fig. 5d), and maximal respiration rate (Fig. 5e), which are used to determine the amount of extra ATP that can be synthesized by mitochondrial respiration in cases of a sudden increase in the cellular energy demand^39^. In addition, overall ATP production from endometrial stem cells was significantly enhanced by PM exposure (Fig. 5f). Because lactate and protons are produced in cells during glycolysis^40,41^, we evaluated the glycolytic activity of endometrial stem cells with or without PM exposure by performing real-time analysis of the extracellular acidification rate (ECAR) in cell culture supernatants. A schematic of the procedure for real-time analysis of glycolysis in endometrial stem cells using the Seahorse XF analyzer is shown in Fig. 5g. 2-Deoxyglucose (2-DG) was added to block glycolysis and therefore provide a baseline ECAR measurement^42^. Rotenone (an inhibitor of complex I in the electron transport chain) and antimycin A (an inhibitor of complex III in the electron transport chain) were added to block mitochondrial respiration completely; thus, no further oxygen consumption occurred, suggesting the occurrence of nonmitochondrial respiration^43^. Real-time analysis of the glycolytic rate showed that PM-treated stem cells exhibited higher maximal glycolysis rates than untreated stem cells (Fig. 5g). PM exposure also markedly increased basal glycolysis (Fig. 5h) and compensatory glycolysis (Fig. 5i). These results suggest that exposure to high levels of PM significantly accelerates both aerobic (mitochondrial respiratory) and anaerobic (cytosolic glycolysis) energy production, which has been shown to lead to subsequent acceleration of cellular aging in multiple types of stem cells^44–46^.

### SERPINB2 and its related signaling pathways are aberrantly activated in endometrial stem cells in response to PM exposure

**Fig. 6:**
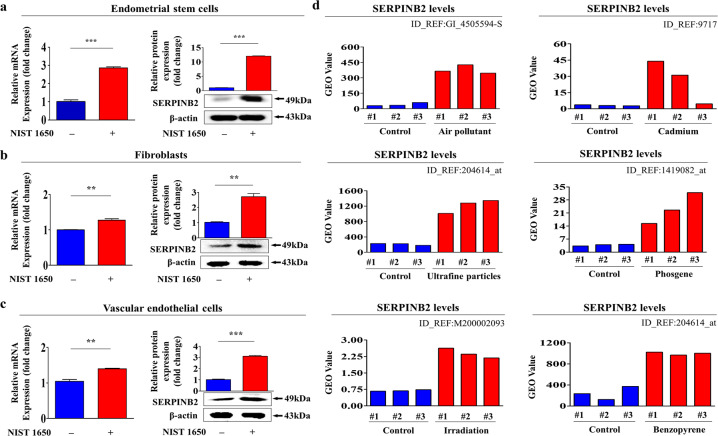
PM exposure stimulates SERPINB2 expression in endometrial stem cells in vitro. We hypothesized that SERPINB2 could act as a key mediator of PM-induced toxic effects in endometrial stem cells. Endometrial stem cells (**a**), fibroblasts (**b**), and vascular endothelial cells (**c**) were treated with or without PM (25 μg/ml) for 72 h. Real-time PCR and western blotting were performed to confirm the increase in SERPINB2 expression by PM exposure in vitro. The GEO database was also analyzed to further verify the correlations between the increased SERPINB2 level and exposure to air pollutants (**d**). β-Actin was used as the internal control. The bar graphs show the average of three independent experiments. Significant differences are presented. *p < 0.05, **p < 0.005, and ***p < 0.001 (two-sample *t*-test).

In our previous studies, we found that SERPINB2, also called plasminogen activator inhibitor 2 (PAI-2), could serve as an effective marker for predicting toxicity in response to multiple hazardous substances in both cord blood-derived stem cells^21^ and various types of cancer stem cells^47^. In this context, to determine whether SERPINB2 can act as a key mediator of PM-induced toxic effects in endometrial stem cells, we investigated the expression profile of SERPINB2 in endometrial stem cells with or without PM exposure using real-time PCR and western blotting. Both the mRNA and protein levels of SERPINB2 were markedely increased in endometrial stem cells following PM treatment. Importantly, we found that PM exposure affected the expression levels of SERPINB2 more profoundly in endometrial stem cells (Fig. 6a) than in terminally differentiated somatic cells, such as dermal fibroblasts (Fig. 6b) and vascular endothelial cells (Fig. 6c). We also analyzed the GEO database to further evaluate positive correlations between the SERPINB2 level and toxicity. Indeed, SERPINB2 expression was significantly increased in response to various types of toxic exposures, such as air pollution, fine particulate matter, radiation, and toxicants (Fig. 6d). In addition, to investigate whether enhanced activity of SERPINB2-associated signaling pathways was positively correlated with exposure to various toxicants, we analyzed various gene expression profiles and their signaling interactions using Ingenuity Pathway Analysis (IPA) software. Positive downstream regulators of SERPINB2, namely, AR, FGF, FGF4, and NT-4, were suppressed in toxicant (TCDD)-exposed cells (Supplementary Fig. 3a). Similarly, positive downstream regulators of SERPINB2, namely, EGF, FGF7, IGFBP4, and IGFBP6, were suppressed in cells in response to a cytotoxic agent (DMSO) (Supplementary Fig. 3b). These results suggest that SERPINB2 can serve as a reliable regulator of reproductive toxicity in human endometrial stem cells.

### SERPINB2 depletion significantly attenuates various PM-induced inhibitory effects on endometrial stem cells

**Fig. 7:**
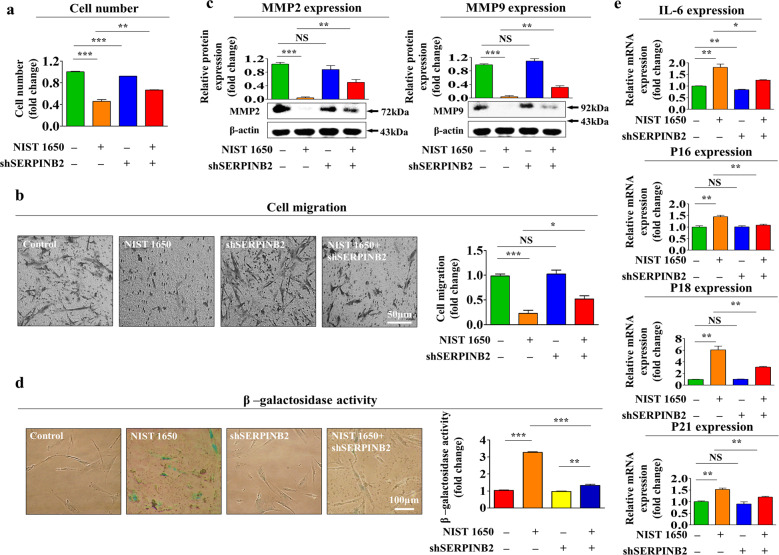
SERPINB2 mediates the inhibitory effect of PM exposure on the self-renewal, migration, and replicative senescence of endometrial stem cells in vitro. Schematic representation showing the functions of SERPINB2 as a gene that regulates PM-induced effects in endometrial stem cells. Endometrial stem cells were transfected with shRNA targeting SERPINB2 and were treated with or without PM (25 μg/ml) for 72 h; subsequent changes in cell viability were measured by an MTT assay (**a**). The ability of SERPINB2 depletion to abolish the PM-induced inhibitory effects on the migratory capacity of endometrial stem cells was analyzed via a Transwell assay (**b**) and western blotting with anti-MMP-2 and anti-MMP-9 antibodies (**c**). The attenuating effects of SERPINB2 depletion on PM-induced replicative senescence were evaluated by measuring SA-β-Gal activity (**d**) and the expression levels of the senescence markers IL-6, p16, p18, and p21 (**e**). β-Actin was used as the internal control. The bar graphs show the average of three independent experiments. Significant differences are presented. *p < 0.05, **p < 0.005, and ***p < 0.001 (two-sample *t*-test).

**Fig. 8:**
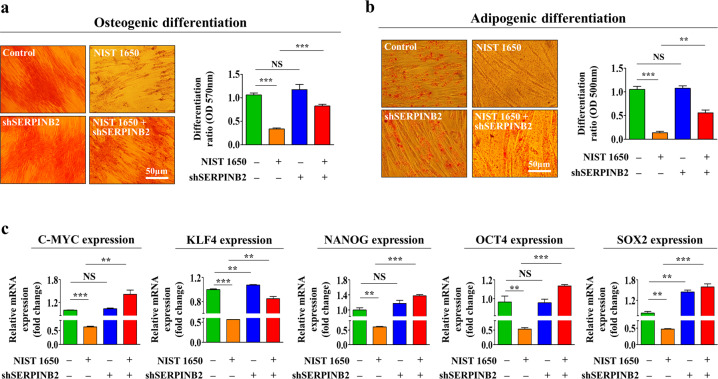
SERPINB2 mediates the inhibitory effect of PM exposure on the multilineage differentiation potential and pluripotency of endometrial stem cells. Endometrial stem cells were transfected with a shRNA targeting SERPINB2 and treated with or without PM (25 μg/ml); subsequent changes in adipocyte (**a**) and osteoblast (**b**) differentiation were evaluated by oil red O and alizarin red staining, respectively. The relative quantification of calcium mineral content and lipid droplet formation in differentiated cells was evaluated by measuring the absorbance at 500 nm and 570 nm, respectively. The attenuating effects of SERPINB2 depletion on PM-induced suppression of the pluripotency-related genes C-MYC, KLF4, NANOG, OCT4, and SOX2 were analyzed by real-time PCR (**c**). The bar graphs show the average of three independent experiments. Significant differences are presented. *p < 0.05, **p < 0.005, and ***p < 0.001 (two-sample *t*-test).

To determine whether SERPINB2 can mediate the PM-induced inhibitory effects on the various beneficial functions of endometrial stem cells, SERPINB2 was stably knocked down by transfecting cells with a shRNA targeting SERPINB2 (Supplementary Fig. 4a–c). Importantly, as shown in Fig. 7a, the PM-induced inhibitory effects on the self-renewal capacity of endometrial stem cells were significantly attenuated by SERPINB2 depletion. After SERPINB2 knockdown, the PM-induced inhibitory effects on the cell migration potential across the membrane were markedly decreased (Fig. 7b). The PM-induced inhibitory effects on the expression of MMP-2/9, which play key roles in regulating cell migration, were successfully attenuated by SERPINB2 depletion (Fig. 7c). Moreover, PM-induced replicative senescence (as indicated by enhanced SA-β-Gal activity) was clearly attenuated by SERPINB2 knockdown (Fig. 7d). Consistent with these results, the expression of the senescence markers p16, p18, p21, and IL-6, which was elevated in response to PM exposure, was significantly reduced by SERPINB2 depletion (Fig. 7e). In addition, the PM-induced inhibitory effects on the multilineage differentiation potential toward the adipocyte (Fig. 8a) and osteoblast (Fig. 8b) lineages in vitro were successfully attenuated by SERPINB2 depletion. The PM-induced suppressive effects on the expression of various pluripotency-associated factors, such as C-MYC, KLF4, NANOG, OCT4, and SOX2, were also significantly reversed by SERPINB2 knockdown (Fig. 8c). These results suggest that SERPINB2 might play a crucial role in mediating the PM-induced inhibitory effects on the various beneficial functions of endometrial stem cells, such as their self-renewal, replicative senescence, migration, and differentiation capacity.

### Proteome Profiler analysis of PM-induced suppression of the expression of various proteins and their interconnected signaling networks

**Fig. 9:**
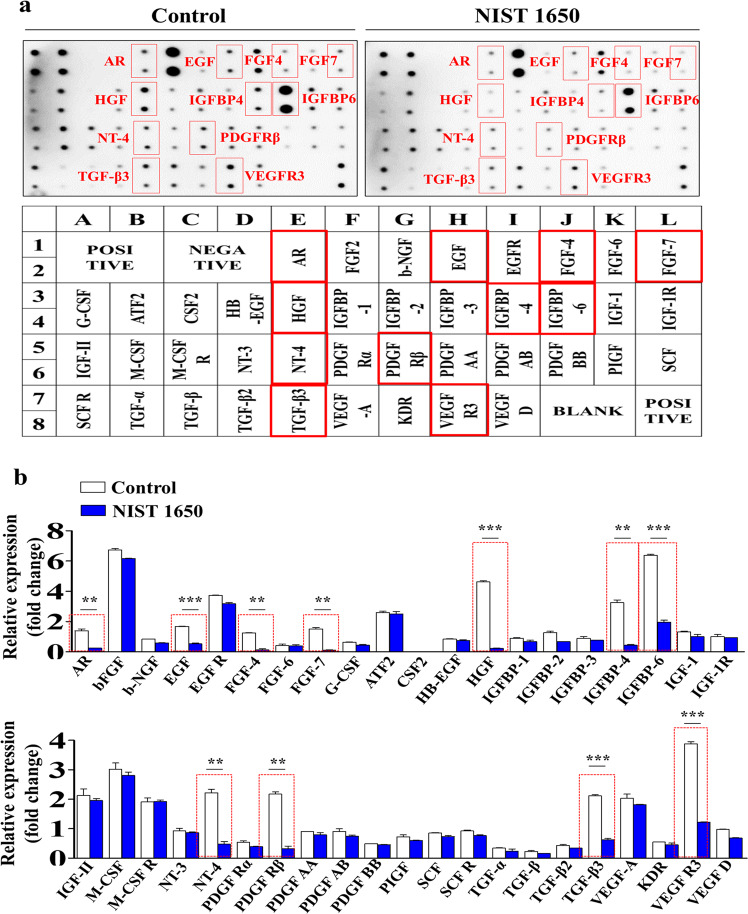
PM exposure suppresses the secretion of various growth factors or cytokines that are related to toxicity networks. Human growth factor antibody array analysis was performed using PM-treated or untreated protein samples. The membrane was printed with antibodies against 40 growth factors, cytokines, and receptors. The levels of eleven growth factors (AR, HGF, NT-4, TGF-β3, EGF, IGFBP4/6, PDGFR β, VEGFR3, and FGF4/7) were significantly decreased in the PM-treated protein samples (**a**, **b**). The bar graphs show the average of three independent experiments. Significant differences are presented. *p < 0.05, **p < 0.005, and ***p < 0.001 (two-sample *t*-test).

**Fig. 10:**
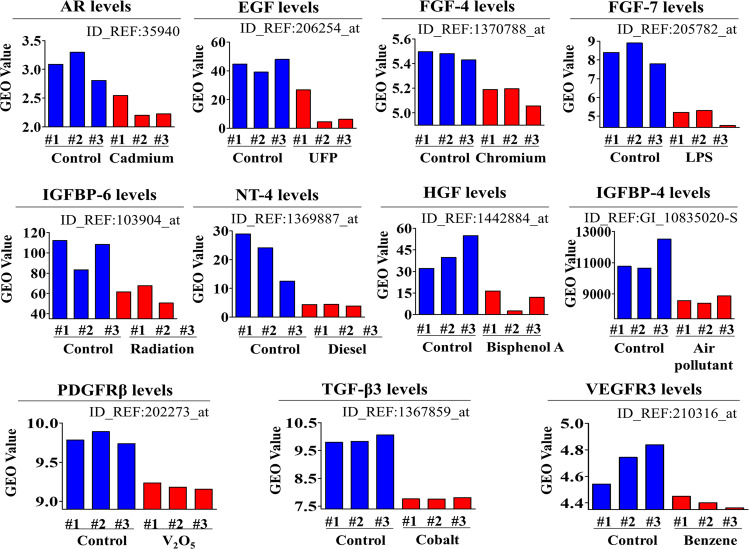
The correlations between the eleven downregulated growth factors and exposure to various toxicants. The GEO database was analyzed to further verify the correlations between the eleven downregulated growth factors and exposure to various toxicants.

We hypothesized that PM exposure can suppress the expression of various growth factors or cytokines in endometrial stem cells, which in turn could be at least partially responsible for the subsequent PM-induced inhibitory effects on multiple beneficial functions of stem cells. Therefore, to identify major proteins whose expression was suppressed by PM exposure, we analyzed the effects of PM on the expression of multiple cytokines or growth factors using antibody arrays. The expression levels of eleven growth factors, namely, the androgen receptor (AR), hepatocyte growth factor (HGF), neurotrophin-4 (NT-4), transforming growth factor beta-3 (TGF-β3), epidermal growth factor (EGF), insulin-like growth factor binding protein 4 and 6 (IGFBP4/6), the platelet-derived growth factor receptor beta (PDGFR β), vascular endothelial growth factor receptor 3 (VEGFR3), and fibroblast growth factor 4 and 7 (FGF4/7), were substantially reduced in endometrial stem cells by PM exposure (Fig. 9a, b). Consistent with these results, GEO database analysis also revealed that the expression levels of these eleven most prominent factors also underwent corresponding changes in response to toxic exposures of various types, such as exposure to air pollutants, toxicants, radiation, and chemicals; however, these changes were not observed in the control groups (Fig. 10). Next, to further investigate whether these eleven most prominent factors were correlated with the signaling pathways regulating the self-renewal capacity, we analyzed various gene expression profiles and their signaling interactions using IPA software. Negative regulators of HGF, such as MET (Z-score = −2.544, *p* value = 1.99E−02), MIF (Z-score = −2.273, *p* value = 3.99E−02), and 1KBKB (Z-score = −2.434, *p* value = 6.12E−04), were inhibited in toxicant (bisphenol A)-exposed cells (Supplementary Fig. 5a). Negative regulators of PDGFRB, such as TP73 (Z-score = −1.044, *p* value = 1.08E−11), HIF3A (Z-score = −1.041, *p* value = 5.24E−04), and AHR (Z-score = −0.782, *p* value = 6.05E−04), were inhibited in toxicant (imidacloprid)-exposed cells (Supplementary Fig. 5b). Negative regulators of IGFBP4, such as F2 (Z-score = −4.016, *p* value = 2.02E−12) and TP53 (Z-score = −5.247, *p* value = 1.60E−02), were inhibited in toxicant (topotecan)-exposed cells (Supplementary Fig. 5c). Negative regulators of IGFBP6, such as RARA (Z-score = −3.828, *p* value = 6.72E−02), IL-6 (Z-score = −4.289, *p* value = 2.80E−09), and NFKB1A (Z-score = −2.192, *p* value = 2.02E−07), were inhibited in toxicant (docetaxel)-exposed cells (Supplementary Fig. 5d). In addition, using GeneMANIA software, we evaluated whether these eleven factors were interconnected with signaling networks governing pluripotency, migratory capacity, or self-renewal ability. To assess functional correlations, datasets were filtered with various criteria, such as coexpression, colocalization, and genetic interactions. The results indicated substantial correlations between these eleven prominent factors and multiple cellular functions, such as self-renewal, pluripotency, and migratory capacity (Supplementary Fig. 6a, b).

### PM exposure suppresses various beneficial functions and decreases the pluripotency of tissue-resident stem cells in vivo

**Fig. 11:**
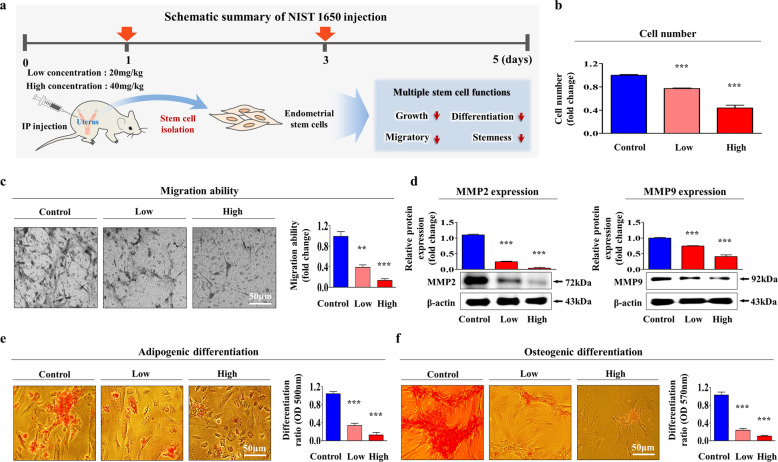
PM exposure significantly inhibits various beneficial functions of endometrial stem cells in vivo. Schematic representation of the experimental protocol as described in the materials and methods section (**a**). Mice were treated two times with a low (20 mg/kg) or high (40 mg/kg) dose of PM or with vehicle (PBS) by intraperitoneal injection. Endometrial stem cells were isolated from the mouse uterine endometrium, and changes in stem cell viability were evaluated by an MTT assay. Stem cell viability (%) was calculated as a percent of the viability of cells treated with vehicle control (**b**). Changes in cell migration were evaluated via a Transwell assay (**c**) and western blotting with anti-MMP-2 and anti-MMP-9 antibodies (**d**). The effects of PM exposure on adipocyte (**e**) and osteoblast (**f**) differentiation in vivo were evaluated by oil red O and alizarin red staining, respectively. The relative quantification of calcium mineral content and lipid droplet formation in differentiated cells was analyzed by measuring the absorbance at 500 nm and 570 nm, respectively. β-Actin was used as the internal control. The bar graphs show the average of three independent experiments. Significant differences are presented. *p < 0.05, **p < 0.005, and ***p < 0.001 (two-sample *t*-test).

Our in vitro results suggested that PM exposure may inhibit the beneficial functions of endometrial stem cells in vivo. Therefore, we further evaluated whether PM exposure has similar effects on endometrial stem cells and their pluripotency in an animal model. Mice were treated twice with a low (20 mg/kg) or high (40 mg/kg) dose of PM by intraperitoneal injection, and endometrial stem cells were then isolated from uterine tissue (Fig. 11a). Consistent with the in vitro results, the in vivo data showed that PM exposure markedly reduced the self-renewal capacity of endometrial stem cells in a dose-dependent manner (Fig. 11b). Additionally, Transwell migration assays revealed the inhibitory effect of PM exposure on the migratory capacity of stem cells in vivo (Fig. 11c). Western blotting with anti-MMP-2 and anti-MMP-9 antibodies was performed to further confirm the inhibitory effect of PM exposure on endometrial stem cell migration (Fig. 11d). Importantly, PM exposure markedly reduced the capacity of the cells to differentiate into adipocytes (Fig. 11e) and osteoblasts (Fig. 11f) in vivo. Consistent with these results, the expression of the pluripotency-related proteins C-MYC, KLF4, NANOG, OCT4, and SOX2 was significantly reduced by PM exposure (Supplementary Fig. 7). In addition, we further determined whether PM exposure inhibits the beneficial functions of other stem cell types, such as adipose tissue-derived stem cells and bone marrow-derived stem cells, in vivo. Similar to the above protocol, mice were treated twice with a low (20 mg/kg) or high (40 mg/kg) dose of PM by intraperitoneal injection, and stem cells were then isolated from adipose tissue (Supplementary Fig. 8a) and bone marrow (Supplementary Fig. 9a). Consistent with the endometrial stem cell results, PM exposure significantly decreased the growth potential (Supplementary Figs. 8b and 9b), migration (Supplementary Figs. 8c/d and 9c/d), and transdifferentiation (Supplementary Figs. 8e–g and 9e–g) capacity of adipose tissue-derived stem cells and bone marrow-derived stem cells in a dose-dependent manner. Taken together, these results suggest that PM exposure may inhibit various beneficial functions of multiple types of tissue-resident stem cells.

## Discussion

Interestingly, the recent decline in female fertility seems to be associated with increased air pollution^48^. Some air pollutants, such as diesel exhaust and heavy metals, can disrupt the endocrine system^49^ and subsequently reduce female reproductive capacity. These endocrine disruptors can dramatically interfere with the hypothalamus-pituitary-thyroid axis and induce various metabolic disorders, including obesity and insulin resistance syndrome, which are associated with increased female infertility^50–52^. Indeed, a small number of studies have revealed that exposure to air pollution could be associated with increased female infertility^53–55^. For instance, Mohallem et al. found a significantly decreased embryo implantation rate and a subsequent reduction in the live birth rate in animals exposed to city air pollutants compared to nonexposed animals^12^. Similarly, Veras et al. observed a significantly increased infertility rate in mice exposed to traffic-related pollution^56^. Furthermore, women who live closer to a major roadway may have an increased rate of infertility compared to women living further from a major roadway, regardless of the specific road type^53^. Furthermore, a higher risk of miscarriage was observed in women exposed to PM with an aerodynamic diameter ≤10 μm during pregnancy^8^. Importantly, a recent epidemiological study conducted by Xue et al. revealed that a reduced fertility rate is associated with pregestational exposure to PM but not with gestational exposure^57^. However, the precise mechanisms underlying the inhibitory effects of PM exposure on female fertility are still a matter of debate.

In humans, the endometrium of the uterine cavity undergoes extensive stromal growth and vascularization in a cyclic manner to facilitate successful embryo implantation, and the entire endometrial surface is completely restored within a few days of menstruation^58^. This remarkable regenerative capacity of the endometrium during each menstrual cycle is necessary for reproductive success. Endometrial stem cells located within the basal layer (stratum basalis) are not shed during each menstrual period, as these are the main cells responsible for cyclical endometrial regeneration^59^. Lucas et al. found that a lack of endometrial stem cells in the basal layer of the uterus seems strongly associated with an increased risk of recurrent pregnancy loss (RPL)^15^. Importantly, significantly decreased numbers of active self-renewing endometrial stem cells were found in 42% of patients experiencing recurrent miscarriage, while such a decrease was found in only 11% of normal control patients^15^. Due to this close relationship between resident endometrial stem cells and endometrial regeneration, it is reasonable to hypothesize that a poor quality of endometrial stem cells may reduce the successful implantation rate and subsequent pregnancy outcomes. Consistent with these observations, Lucas et al. found that senescent endometrial stem cells promote inflammation and trigger a chronic autoinflammatory response, which might be associated with an increased risk of recurrent miscarriage^60^. In this context, intensive investigation of resident stem cells may provide important insights into the major causes of uterine defect-related infertility. In particular, new challenging questions have arisen regarding the potential effect of PM on endometrial stem cells and its precise molecular mechanisms underlying the relatively low fertility rate in response to PM exposure. We therefore hypothesized that exogenous PM exposure directly damages the uterine endometrium by suppressing various endometrial stem cell functions and, as a consequence, reduces favorable pregnancy outcomes.

Consistent with our hypothesis, PM exposure significantly inhibited various beneficial functions, such as the self-renewal, reproductive senescence, migration, multilineage differentiation potential, and metabolic phenotypes, of endometrial stem cells in vitro (Figs. 1–5) and in vivo (Fig. 11a–g). Interestingly, metabolic activities (mitochondrial oxidative phosphorylation and glycolysis) were significantly enhanced by PM exposure (Fig. 5a–i). In contrast, recent studies have suggested that iPSCs (induced pluripotent stem cells) preferentially switch from oxidative phosphorylation to aerobic glycolysis, which may be required for their self-renewal capacity and pluripotency^61^. Consistent with these results, enhancing glycolytic dependency by regulating glycolytic substrates or metabolic mediators can increase the reprogramming efficiency of differentiated cells, whereas disrupting glycolysis by exposure to various metabolic inhibitors has the opposite effect^62–64^. However, the efficiency of glycolysis-associated reprogramming varies among cell types, and different cell types use various metabolic routes to attain a reprogrammed pluripotent state^65^. In this context, it is currently difficult to postulate that dioxin-induced glycolytic activity may not be associated with the differentiation capacity and pluripotency of endometrial stem cells. The enhanced glycolytic activity induced by dioxin exposure is very likely related to dioxin-induced toxic responses. Several previous studies found that dioxin (TCDD) exposure can enhance glycolysis in an organ-specific manner as part of the toxic response. For instance, Moore et al. observed that dioxin increased the mRNA levels of various glycolytic genes in the liver^66^. Wu et al. also found that AMPK treatment significantly enhanced glycolysis as an adaptive response to oxidative stress in human skin fibroblasts^67^.

In addition, PM exposure markedly suppressed the expression of eleven growth factors or cytokines, namely, AR, EGF, FGF4/7, HGF, IGFBP4/6, NT-4, PDGFR β, TGF-β3, and VEGFR3 (Fig. 9a–b). In addition, GEO database analysis revealed that the relative expression levels of these eleven most prominent factors underwent corresponding changes in response to various toxic exposures (Fig. 10). These results suggest that the PM-induced inhibitory effects on endometrial stem cells are due at least in part to the decreased expression of various cytokines or growth factors, which can regulate multiple biological functions, including apoptosis, migration, pluripotency, and self-renewal. In addition, the PM-induced inhibitory effects on stem cell growth (Fig. 7a), migration (Fig. 7b, c), replicative senescence (Fig. 7d, e) and pluripotency (Fig. 8a–c) were markedly attenuated by SERPINB2 knockdown. These results indicate that SERPINB2 may serve as a functional regulator to mediate the various inhibitory effects of PM exposure on various endometrial stem cell functions. Consistent with this hypothesis, recent studies have revealed that increased SERPINB2 expression significantly reduces the self-renewal capacity and differentiation potential of stem cells^20^. Abnormal expression of SERPINB2 has also been correlated with poor prognosis and tumor progression in multiple cancer types, such as bladder^68^, colorectal^69^, endometrial^70^ and ovarian^71^ cancers. In addition, Qin Hu et al. found that dioxin exposure increased SERPINB2 expression in human epidermal keratinocytes^72^. These results suggest that SERPINB2 can serve as a key regulator that mediates toxic responses to various potentially hazardous substances. In our previous study, we also observed that increased SERPINB2 expression significantly reduced the migratory capacity, multilineage differentiation potential, and self-renewal ability of human umbilical cord blood-derived stem cells^21^. In addition, the stromal cells of the endometrium undergo transformation into more rounded and highly specialized secretory epithelial-like cells, termed decidualized stromal cells. These cells provide the nutrients and maternal immune tolerance essential for successful blastocyst implantation and subsequent placenta formation. For these reasons, it is very important to evaluate the effect of PM exposure on the decidualization process of endometrial cells. Although the effects of PM exposure on the decidualization of endometrial cells have not yet been investigated, several studies have revealed that PM exposure decreases the placental mass, size, and surface area^73^. These results may indicate that PM exposure possibly impairs trophoblast invasion and placental angiogenesis by inhibiting the decidualization of endometrial cells. It is therefore assumed that investigating the effects of PM exposure on the decidualization of endometrial cells in vitro and in vivo in our future study would be sufficient to resolve this major uncertainty.

**Fig. 12:**
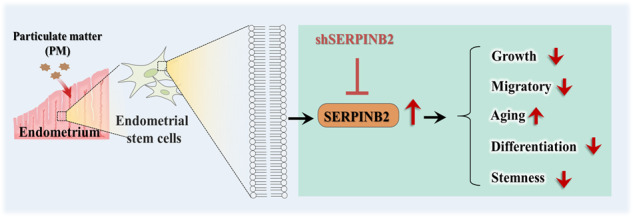
Schematic summary of this study. PM exposure significantly inhibited various beneficial functions of endometrial stem cells, such as their self-renewal, transdifferentiation, and migratory capacity, in vitro and in vivo through its target gene SERPINB2, which has recently been shown to be involved in multiple stem cell functions.

Taken together, these findings suggest that PM-induced exposure directly damages the uterine endometrium and consequently reduces the rate of favorable pregnancy outcomes by inhibiting various functions of endometrial stem cells. To the best of our knowledge, the present study is the first to focus on the direct effects of PM exposure on the regenerative capacity of endometrial stem cells and to provide evidence that SERPINB2 may act as a key mediator of PM-induced toxic effects in endometrial stem cells (summarized in Fig. 12). This knowledge could provide a better understanding of the mechanisms underlying the relatively low female fertility rate resulting from PM exposure. Furthermore, our findings may facilitate the development of promising therapeutic strategies for improving reproductive outcomes in infertile women.

## Supplementary information

Supplementary figures and legends
