# Supplementary material for: The impact of fine particulate matter (PM) on various beneficial functions of human endometrial stem cells through its key regulator SERPINB2
Source: Exp Mol Med. 2021 Dec 2;53(12):1850–65. doi: 10.1038/s12276-021-00713-9 (PMC8741906; doi:10.1038/s12276-021-00713-9)
Supplement: Supplementary file 1 — Supplementary figures and legends [file 12276_2021_713_MOESM1_ESM.pdf]

Supplementary fig. 1

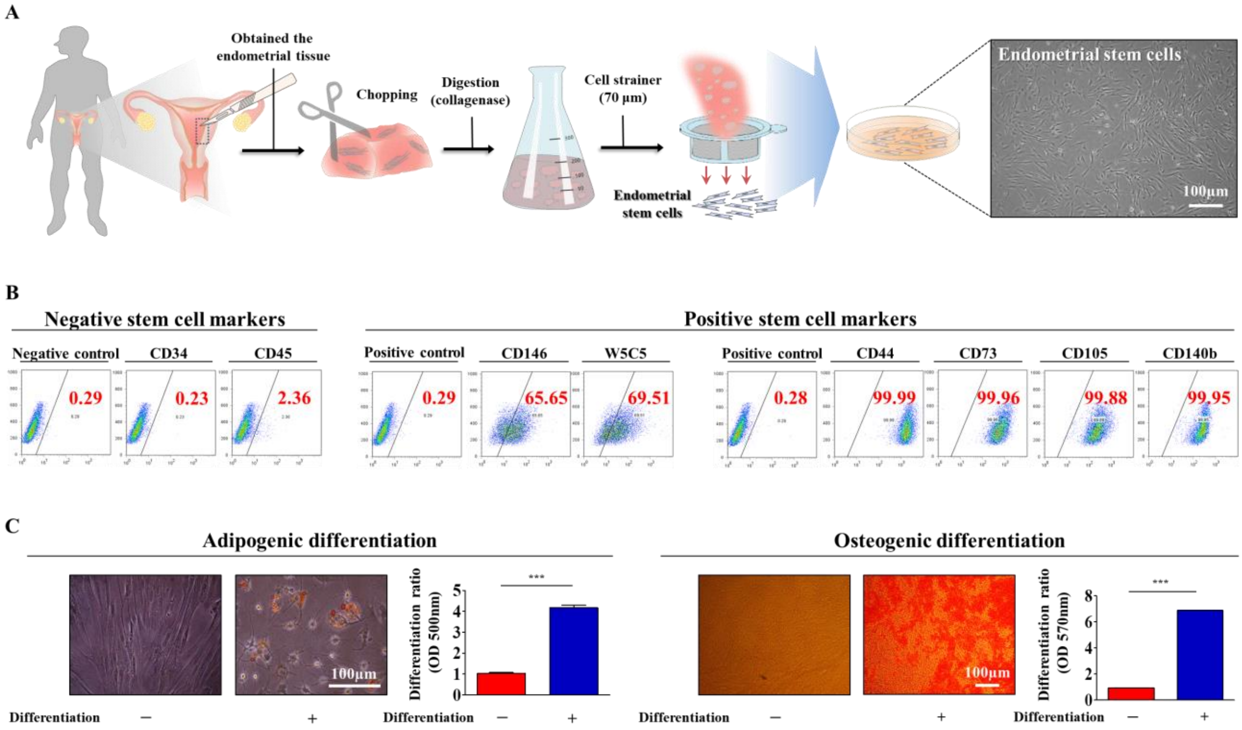

Supplementary Fig. 1 Isolation and characterization of human endometrial stem cells from uterine tissues. Human endometrial stem cells were observed under a phase-contrast microscope to evaluate their overall cell morphology **(a)**. The isolated endometrial stem cells were positive for multiple surface markers CD44, CD73, CD105, CD140b, CD146, and W5C5 as well as negative for the several hematopoietic markers CD34 and CD45 **(b)**. Their ability to differentiate into adipocytes and osteoblasts was determined by oil red O staining and alizarin red staining, respectively. The relative quantification of calcium mineral content and lipid droplet formation within the differentiated cells was performed by measuring the absorbance at 500 nm and 570 nm, respectively **(c)**. Significant differences are presented. $*p < 0.05$, $**p < 0.005$, and $***p < 0.001$ (two-sample t-test).

Supplementary fig. 2

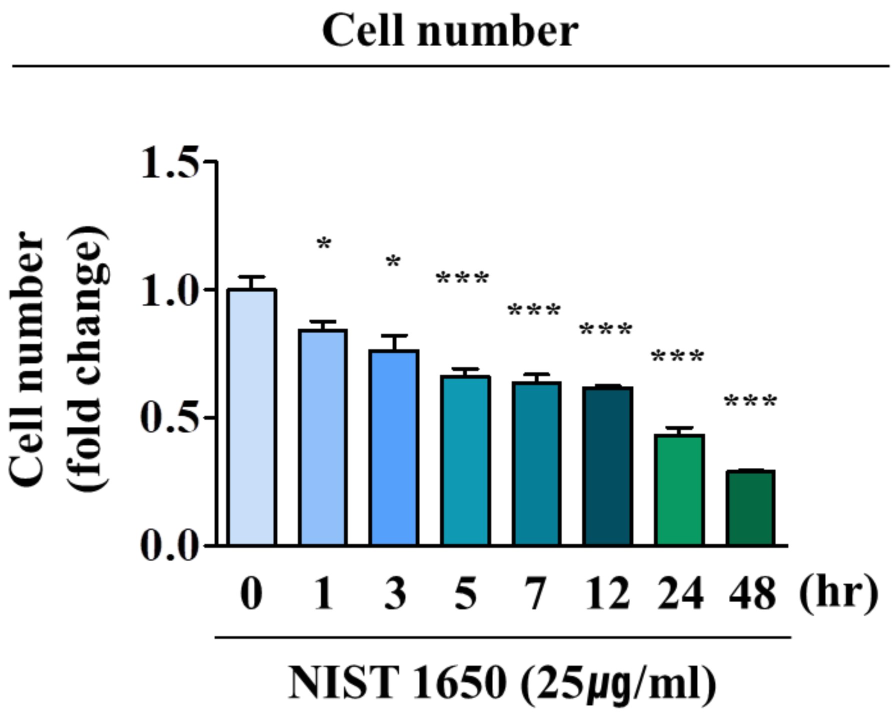

Supplementary Fig. 2 PM treatment significantly decreased the self-renewal capacity of endometrial stem cells in a time-dependent manner. The inhibition of endometrial stem cell self-renewal by treatment with various treatment times (1, 3, 5, 7, 12, 24, 48hr) of PM (25 ug/ml) was evaluated at 72 h by an MTT assay. Stem cell viability (%) was calculated as a percent of the viability of cells treated with the vehicle control. Significant differences are presented. *$p < 0.05$, **$p < 0.005$, and ***$p < 0.001$ (two-sample t-test).

Supplementary fig. 3

A

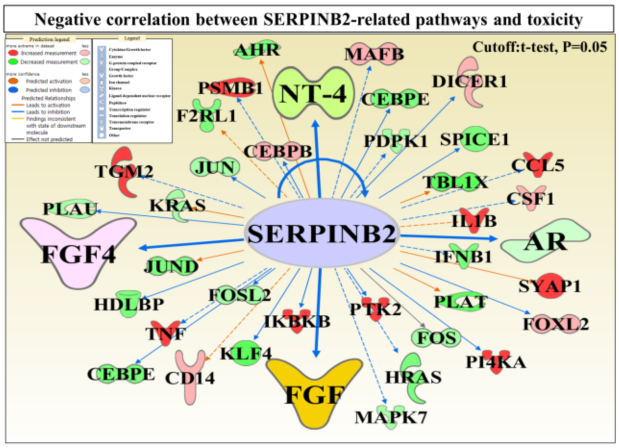

B

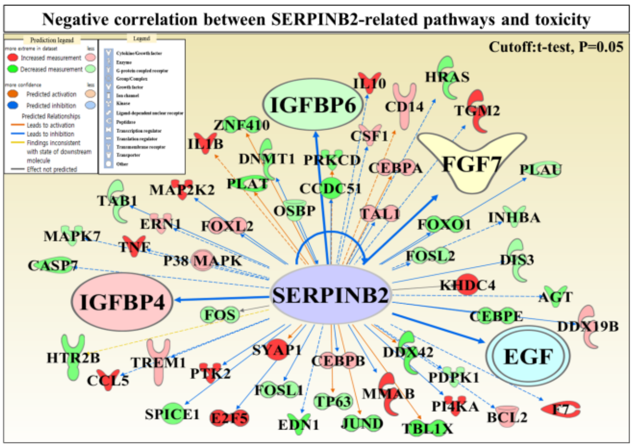

Target gene	Predicted activation group	Predicted activation state	Activation z-score	P-value of overlap
AR	Toxicant treated cells (vs non-treated cells)	Inhibition	-3.995	1.05E-23
FGF	Toxicant treated cells (vs non-treated cells)	Inhibition	-3.995	1.05E-23
FGF4	Toxicant treated cells (vs non-treated cells)	Inhibition	-3.995	1.05E-23
NT-4	Toxicant treated cells (vs non-treated cells)	Inhibition	-3.995	1.05E-23

Target gene	Predicted activation group	Predicted activation state	Activation z-score	P-value of overlap
EGF	Toxicant treated cells (vs non-treated cells)	Inhibition	-3.351	6.18E-15
FGF7	Toxicant treated cells (vs non-treated cells)	Inhibition	-3.351	6.18E-15
IGFBP4	Toxicant treated cells (vs non-treated cells)	Inhibition	-3.351	6.18E-15
IGFBP6	Toxicant treated cells (vs non-treated cells)	Inhibition	-3.351	6.18E-15

Supplementary Fig. 3 SERPINB2-associated signaling pathways are correlated with various toxicant exposures. Differentially expressed genes from toxicant (TCDD)-exposed cells and nontreated cells (GSE69851) **(a)** or cytotoxic agent (DMSO)-treated cells and nontreated cells (GSE69851) **(b)** were analyzed using ingenuity pathway analysis (IPA) software (http://www.ingenuity.com) to predict the activation state of the toxicant-related signaling pathways.

Supplementary fig. 4

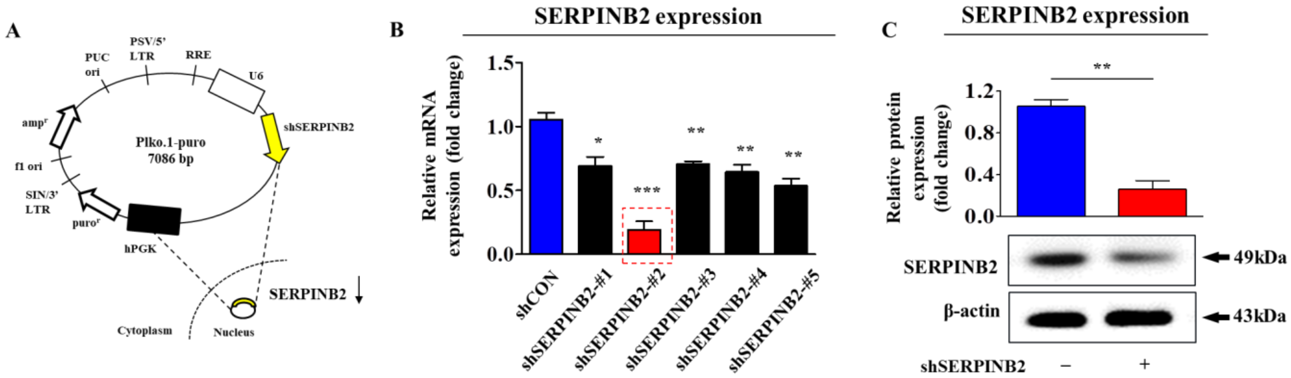

Supplementary Fig. 4 Knockdown efficacy of shRNAs targeting SERPINB2 in endometrial stem cells. Endometrial stem cells were stably transduced with shRNA #1, #2, #3, #5, or #4, which target SERPINB2, or with a non-targeting control shRNA **(a)**. SERPINB2 shRNA construct #2, hereafter referred to as SERPINB2 shRNA, was the most effective. The successful knockdown of SERPINB2 expression was verified based on RNA **(b)** and protein expression levels **(c)** in endometrial stem cells. β-actin was used as the internal control. Significant differences are presented. $^*p < 0.05$, $^{**}p < 0.005$, and $^{***}p < 0.001$ (two-sample t-test).

Supplementary fig. 5

A

Negative correlation between HGF-related pathways and toxicity

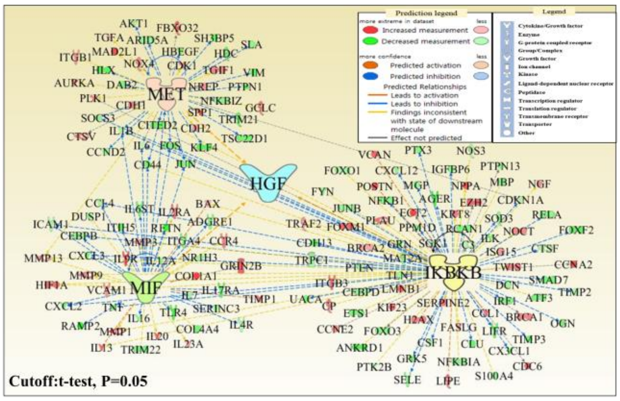

Signaling pathways	Predicted activation group	# of Target molecules in dataset	Predicted activation state	Activation z-score	P-value of overlap
**MET**	Toxicant treated cells (vs non-treated cells)	115	Inhibition	-2.544	1.99E-02
**MIF**	Toxicant treated cells (vs non-treated cells)	44	Inhibition	-2.273	3.99E-02
**1KBKB**	Toxicant treated cells (vs non-treated cells)	46	Inhibition	-2.434	6.12E-04

B

Negative correlation between PDGFRB-related pathways and toxicity

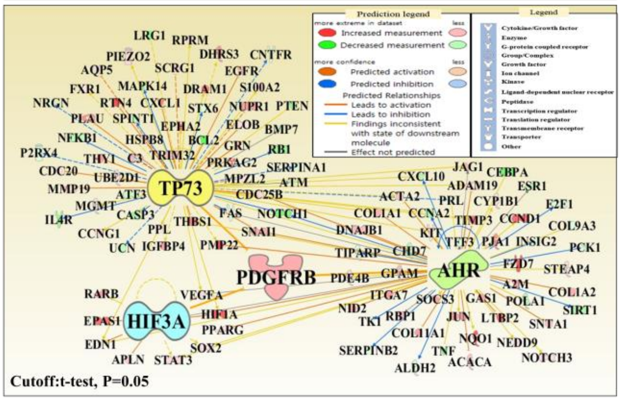

Signaling pathways	Predicted activation group	# of Target molecules in dataset	Predicted activation state	Activation z-score	P-value of overlap
**TP73**	Toxicant treated cells (vs non-treated cells)	154	Inhibition	-1.044	1. 08E-11
**HIF3A**	Toxicant treated cells (vs non-treated cells)	9	Inhibition	-1.041	5.24E-04
**AHR**	Toxicant treated cells (vs non-treated cells)	110	Inhibition	-0.782	6.05E-04

C

Negative correlation between IGFBP4-related pathways and toxicity

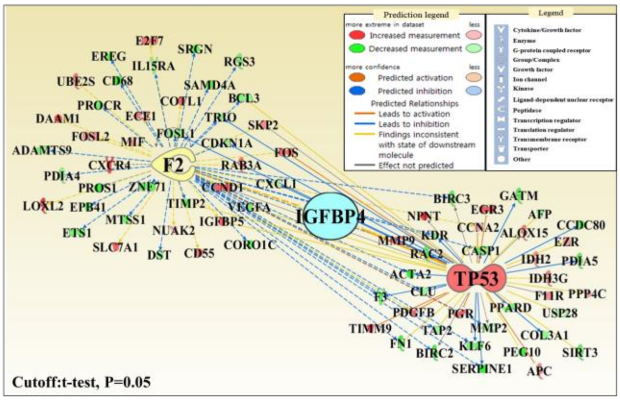

Signaling pathways	Predicted activation group	# of Target molecules in dataset	Predicted activation state	Activation z-score	P-value of overlap
**F2**	Toxicant treated cells (vs non-treated cells)	226	Inhibition	-4.016	2.02E-12
**TP53**	Toxicant treated cells (vs non-treated cells)	846	Inhibition	-5.247	1.60E-22

D

Negative correlation between IGFBP6-related pathways and toxicity

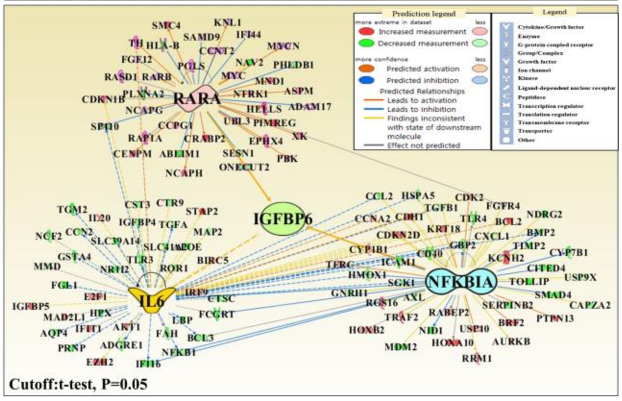

Signaling pathways	Predicted activation group	# of Target molecules in dataset	Predicted activation state	Activation z-score	P-value of overlap
**RARA**	Toxicant treated cells (vs non-treated cells)	68	Inhibition	-3.828	6.72E-02
**IL6**	Toxicant treated cells (vs non-treated cells)	345	Inhibition	-4.289	2.80E-09
**NFKB1A**	Toxicant treated cells (vs non-treated cells)	202	Inhibition	-2.192	2.02E-07

Supplementary Fig. 5 The signaling integrity of the several PM-induced prominent factors is correlated with self-renewal capacity. Differentially expressed genes from toxicant-exposed cells and non-treated cells were analyzed using IPA software to predict the activation state (either activated or inhibited) of HGF (GSE69851), PDGFRB (GSE69851), IGFBP4 (GSE116436), or IGFBP6 (GSE60408)-related signaling integrity, respectively **(a-d)**.

Supplementary fig. 6

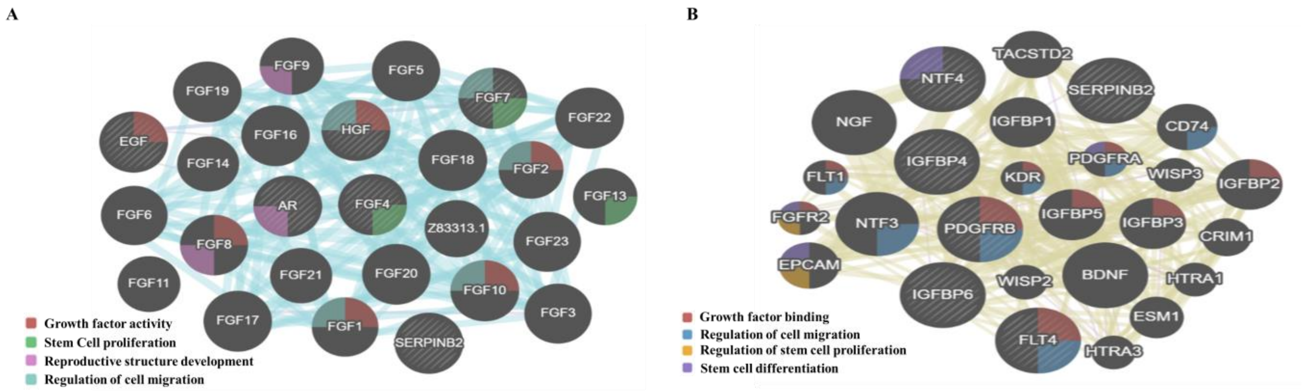

Supplementary Fig. 6 The functional correlation between the PM-induced factors and their signaling networks governing various cellular functions. Signaling network analysis was performed using GeneMANIA (http://www.genemania.org) to predict the connections between PM-induced enhancement of eleven growth factors (AR, HGF, NT-4, TGF-β3, EGF, IGFBP4/6, PDGFR β, VEGFR3, and FGF4/7) and the signaling networks governing self-renewal, pluripotency, and migratory capacity. The results indicate a strong correlation between these eleven prominent factors and multiple cellular functions, such as self-renewal, pluripotency, and migratory capacity **(a-d)**.

Supplementary fig. 7

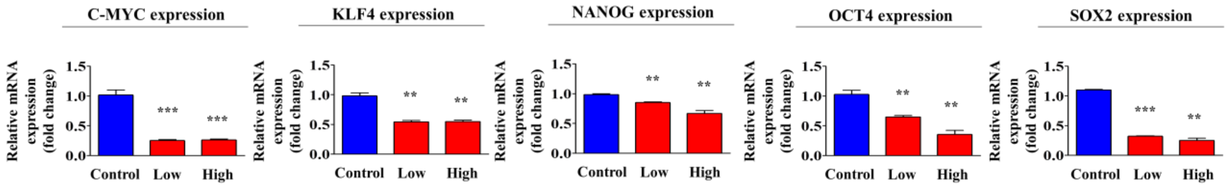

Supplementary Fig. 7 Real-time PCR results revealed changes in the expression of the mouse stem cell markers C-MYC, KLF4, NANOG, OCT4, and SOX2 after PM exposure *in vivo*. Significant differences are presented. *p < 0.05, **p < 0.005, and ***p < 0.001 (two-sample t-test).

Supplementary fig. 8

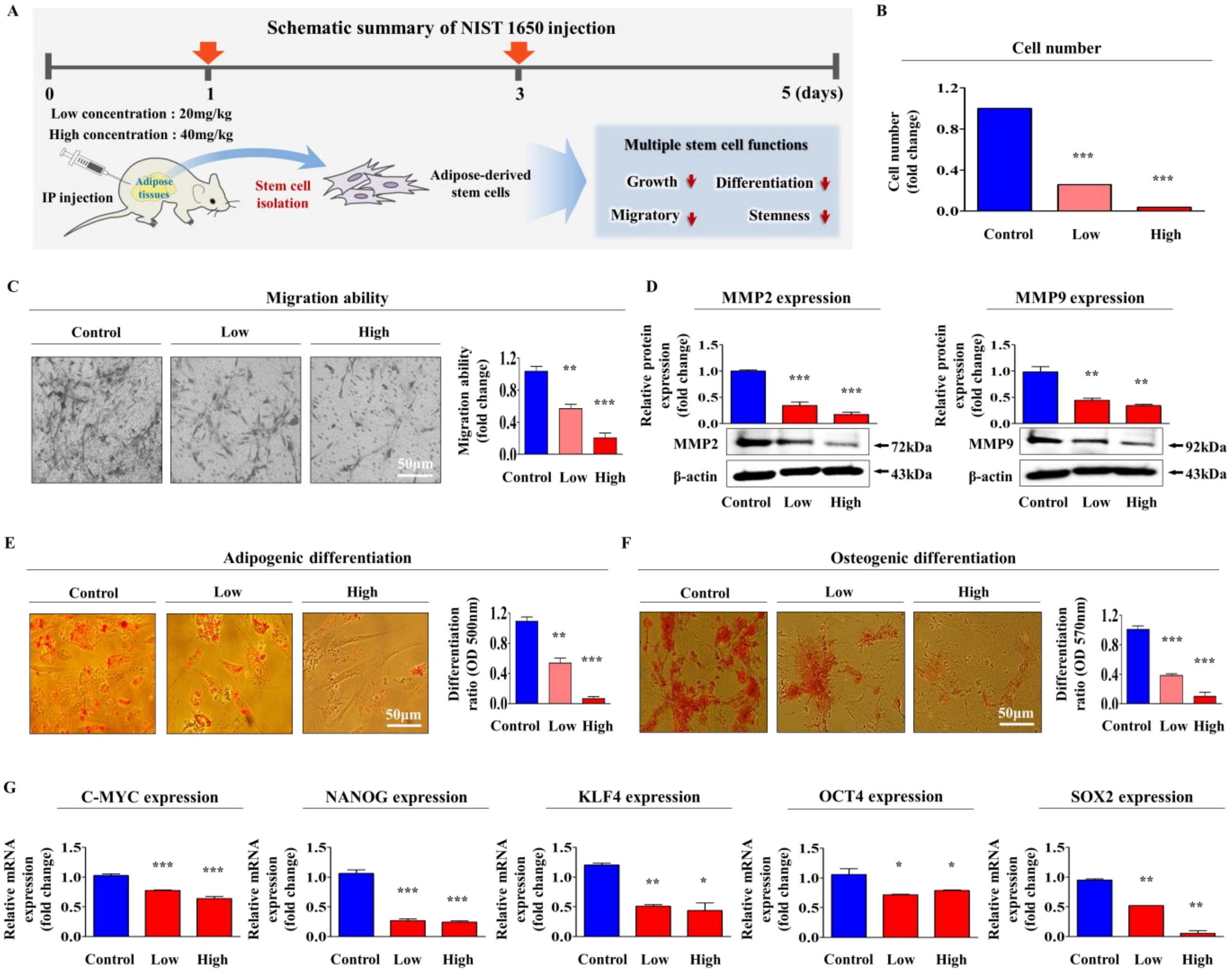

Supplementary Fig. 8 PM exposure significantly inhibits various beneficial functions of adipose tissue-derived stem cells *in vivo*. Schematic representation of the experimental protocol as described in the materials and methods section **(a)**. Mice were intraperitoneally treated two times with low (20 mg/kg) and high (40 mg/kg) dose PM or vehicle (PBS). Adipose tissue-derived stem cells were isolated from mouse adipose tissues, and changes in stem cell viability were evaluated by an MTT assay. Stem cell viability (%) was analyzed as a percent of the vehicle control **(b)**. The changes in cell migration were evaluated via the transwell assay **(c)** and western blotting using MMP-2 and MMP-9 antibodies **(d)**. The effects of PM exposure on adipocyte **(e)** and osteoblast **(f)** differentiation *in vivo* were determined by oil red O and alizarin red staining, respectively. The relative quantification of calcium mineral content and lipid droplet formation within differentiated cells was analyzed by

measuring absorbance at 500 nm and 570 nm, respectively. Real-time PCR results showed the changes in the expression of the mouse stem cell markers C-MYC, KLF4, NANOG, OCT4, and SOX2 after PM exposure *in vivo* **(G)**. β-actin was used as the internal control. Significant differences are presented. *$p < 0.05$, **$p < 0.005$, and ***$p < 0.001$ (two-sample t-test).

Supplementary fig. 9

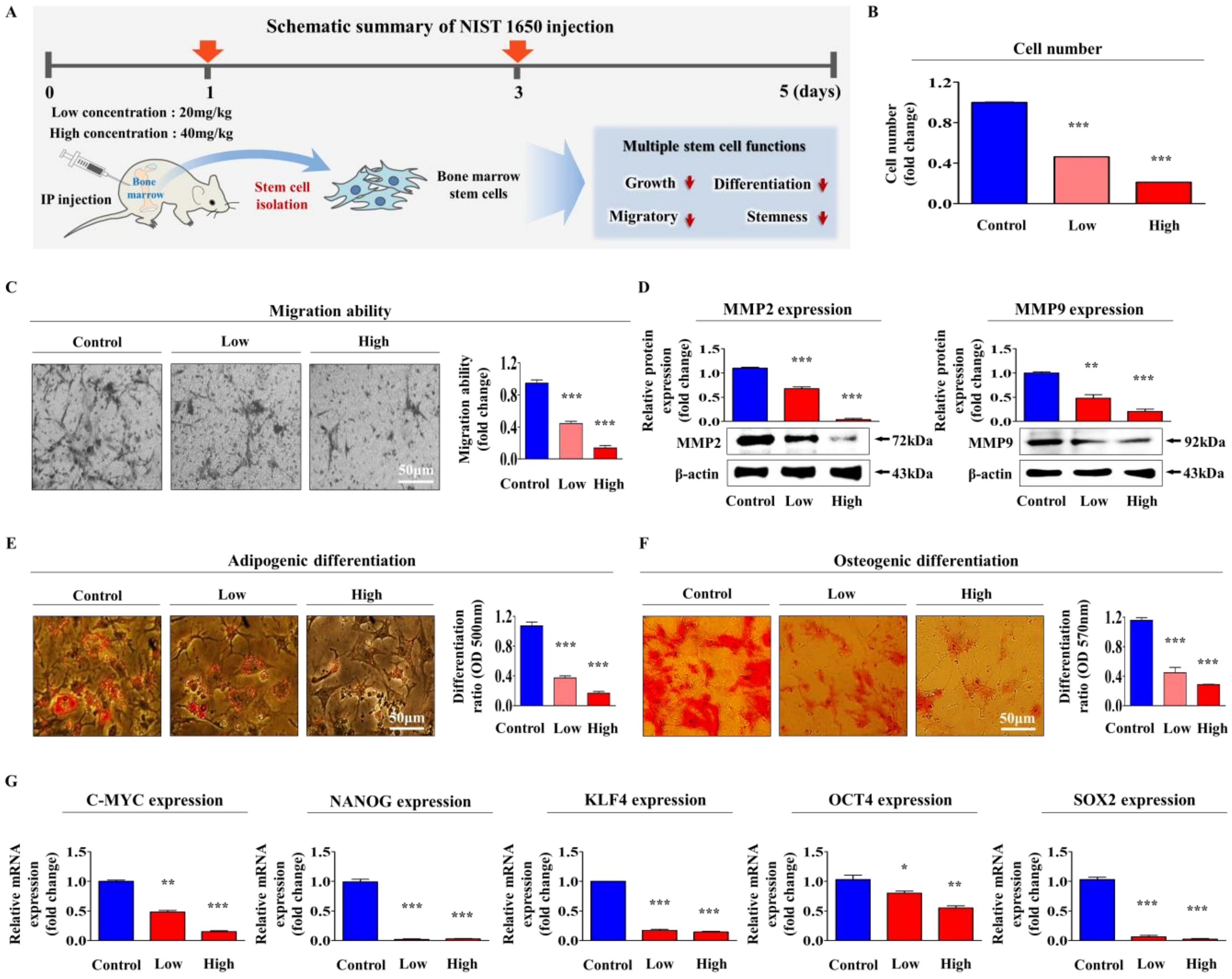

Supplementary Fig. 9 PM exposure significantly inhibits various beneficial functions of bone marrow-derived stem cells *in vivo*. Schematic representation of the experimental protocol as described in the materials and methods section **(a)**. Mice were intraperitoneally treated two times with low (20 mg/kg) and high (40 mg/kg) dose PM or vehicle (PBS). Bone marrow-derived stem cells were isolated from mouse bone marrows, and changes in cell viability were evaluated by an MTT assay. Stem cell viability (%) was analyzed as a percent of the vehicle control **(b)**. The changes in cell migration were evaluated via the transwell assay **(c)** and western blotting using MMP-2 and MMP-9 antibodies **(d)**. The effects of PM exposure on adipocyte **(e)** and osteoblast **(f)** differentiation *in vivo* were evaluated by oil red O and alizarin red staining, respectively. The relative quantification of calcium mineral content and lipid droplet formation within differentiated cells was analyzed by

measuring absorbance at 500 nm and 570 nm, respectively. Real-time PCR results showed the changes in the expression of the mouse stem cell markers C-MYC, KLF4, NANOG, OCT4, and SOX2 after PM exposure *in vivo* **(G)**. β-actin was used as the internal control. Significant differences are presented. *$p < 0.05$, **$p < 0.005$, and ***$p < 0.001$ (two-sample t-test).
